# Intravitreal anti-vascular endothelial growth factor, laser photocoagulation, or combined therapy for diabetic macular edema: A systematic review and network meta-analysis

**DOI:** 10.3389/fendo.2023.1096105

**Published:** 2023-02-02

**Authors:** Jiasheng Chen, Haowei Wang, Weiqiang Qiu

**Affiliations:** ^1^ Department of Ophthalmology, Peking University Third Hospital, Beijing, China; ^2^ Beijing Key Laboratory of Restoration of Damaged Ocular Nerve, Peking University Third Hospital, Beijing, China

**Keywords:** diabetic macular edema, anti-vascular endothelial growth factor, laser photocoagulation, network meta-analysis, combined therapy

## Abstract

**Purpose:**

To conduct a network meta-analysis (NMA) comparing the efficacy of anti-vascular endothelial growth factor (VEGF) therapy alone versus laser photocoagulation (LP) therapy alone or anti-VEGF therapy combined with LP therapy for diabetic macular edema (DME).

**Methods:**

PubMed, Embase, Web of Science, and Cochrane Central Register of Controlled Trials were systematically searched for studies comparing anti-VEGF therapy alone versus LP therapy alone or anti-VEGF therapy combined with LP therapy for DME. Primary outcomes were mean best-corrected visual acuity (BCVA) and central macular thickness (CMT) change. Relevant data were collected and pooled using NMA.

**Results:**

A total of 13 randomized controlled trials were included in our NMA. Anti-VEGF therapy significantly improved BCVA the most compared to the combined (mean difference [MD] = 1.5; 95% confidence interval [CI]: 0.084, 2.7) and LP (MD = 6.3; 95% CI: 5.1, 7.6) therapies at six months, while there was no difference in reducing CMT at six months between the anti-VEGF and combined therapies (MD = -16; 95% CI: -46, 13). At 12 months, no significant difference was found between the anti-VEGF and combined therapy in terms of BCVA (MD = 0.1; 95% CI: -1.7, 1.5) and CMT (MD = 21; 95% CI: -3.0, 44).

**Conclusion:**

There was no significant difference between the anti-VEGF therapy and combined therapy. For the long-term treatment of patients with DME, combined therapy is recommended.

**Systematic review registration:**

https://www.crd.york.ac.uk/prospero/, identifier CRD42022376401.

## Introduction

Diabetic macular edema (DME), a manifestation of diabetic retinopathy (DR) that is diagnosed at any stage of the disease, is defined as retinal oedema and/or thickening, involving, or threatening the fovea. Although the management of diabetes mellitus (DM) has advanced tremendously over the last few decades, DME still accounts for a significant cause of vision loss among patients with DM, and if untreated, can result to blindness. DME affects approximately 7% of patients with DM (1) and represents a substantial public health concern worldwide (2, 3). The prevalence of DME is related to the duration of DM and stage of DR (4).

In recent years, with further understanding of the pathophysiological mechanisms of DME, treatment options for DME have shifted gradually. Laser photocoagulation (LP) was the gold standard treatment for DME prior to the availability of anti-vascular endothelial growth factor (VEGF) treatment (5). The mechanisms of LP include increased oxygen tension and phagocytosis of glial cells and retinal pigment epithelial cells, together with decreased production of vasoactive cytokines (mainly VEGF). LP provides vision stabilization in DME, while the efficacy of providing clinical improvement in patients’ vision seems to be limited (5, 6). Currently, anti-VEGF agents are the first-line treatment option for DME. Ranibizumab, aflibercept, bevacizumab, and pegaptanib have shown significant efficacy in visual improvement in patients with DME in phase II/III clinical trials (7–10). However, anti-VEGF agents cannot treat macular hypoxia; thus, their efficacy is transitory. Additionally, the short half-life of anti-VEGF agents, such as ranibizumab and bevacizumab, in the eyes of 2.75 and 9.8 days, respectively, results in a limited duration of action with consequent high rate of recurrence; thus, requiring frequent injections (11, 12) and imposing a large burden on patients with DME. A combination of anti-VEGF and LP may be more effective than either monotherapy and may reduce the frequency of injections. Additionally, the effectiveness of LP may be improved by LP becoming easier because of the reduction in macular edema caused by anti-VEGF injections. Several studies have evaluated LP as an adjunctive treatment for anti-VEGF agents; however, their conclusions are inconsistent (13–18).

Network meta-analysis (NMA) is a novel data synthesis method that combines direct and indirect evidence from randomized controlled trials (RCTs) using statistical techniques to derive estimates of comparative efficacy (19). Therefore, this study compared the efficacy of anti-VEGF therapy alone, LP therapy alone, or anti-VEGF therapy combined with LP therapy in the treatment of patients with DME within an NMA framework, primarily aimed at assessing the mean best-corrected visual acuity (BCVA) and central macular thickness (CMT) changes.

## Methods

The NMA was strictly conducted in accordance with the Preferred Reporting Items for Systematic Reviews and Meta-analyses (PRISMA) statement (20) and the Cochrane Handbook guidelines (21).

### Search strategy

RCTs evaluating the efficacy of anti-VEGF therapy alone, LP therapy alone, or anti-VEGF therapy combined with LP therapy in the treatment of DME were systematically searched in PubMed, Embase, Web of Science, and the Cochrane Central Register of Controlled Trials from inception to September 11, 2022. The search strategy (**Table S1**) was conducted corresponding to the following terms: “diabetic macular edema,” “anti,” “vascular endothelial growth factor,” “vegf,” “ranibizumab,” “bevacizumab,” “aflibercept,” “pegaptanib,” “laser,” and “photocogulation,” which were connected by and/or in different combinations. The search was restricted to human studies. No publication date or language limitation was imposed when searching for the RCTs. Additionally, reference lists of relevant articles were manually examined to identify potentially relevant studies.

### Inclusion and exclusion criteria

Studies were eligible if they met the following inclusion criteria: (1) RCT; (2) patients/participants with DME; (3) comparison of at least two of the following comparators: anti-VEGF therapy alone, LP therapy alone, anti-VEGF therapy combined with LP therapy; (4) outcome measures, including the mean BCVA and/or CMT change; and (5) follow-up >6 months.

Exclusion criteria were as follows: (1) review articles, case reports, non-RCTs, meta-analyses, and redundant publications; and (2) studies with insufficient data.

Two authors (J C and H W) independently screened the titles and abstracts of the identified articles. All potentially eligible articles were full-text reviewed to evaluate whether they met the inclusion criteria. Any discrepancies were resolved *via* discussion. Unsettled discrepancies were arbitrated by a senior reviewer, Prof. Qiu.

### Data extraction and quality assessment

Two authors (J C and H W) independently extracted data from all the included studies. The extracted data included the first author, publication year, geographic location, study design, interventions (including specific injection plan), follow-up time, and total number of eyes of different interventions, together with the details of outcomes, which included the mean BCVA and CMT change from baseline to 6 and 12 months. If any essential information was required for eligibility assessment or data extraction, the corresponding authors of the included studies were contacted. Logarithm of the minimal angle of resolution (logMAR) was converted into the ETDRS letter form when extracting BCVA data. The Cochrane collaboration tool was used to assess risk of bias (21).

### Statistical analysis

The NMA was performed within a Bayesian framework to synthesize the mean BCVA and CMT changes from baseline to 6 and 12 months across the RCTs. We used R software (version 4.2.1) with gemtc and rjag packages to create forest plots. Statistical heterogeneity was evaluated using the I^2^ statistic: <25%, no heterogeneity; 25–50%, low heterogeneity; 50–75%, moderate heterogeneity; and >75%, high heterogeneity (22). The node-splitting method was used to assess the inconsistency between direct and indirect comparisons in NMA (23). Significant heterogeneity was at p < 0.05. Efficacy of the interventions was evaluated using mean difference (MD) with 95% confidence interval (CI). Additionally, we conducted a ranking analysis based on simulations and calculated the rank’s possibility of establishing a hierarchy of different interventions. We also assessed the potential publication bias by creating the funnel plot and conducting the Egger’s test in the traditional meta-analysis.

## Result

### Study characteristics

**Figure 1:**
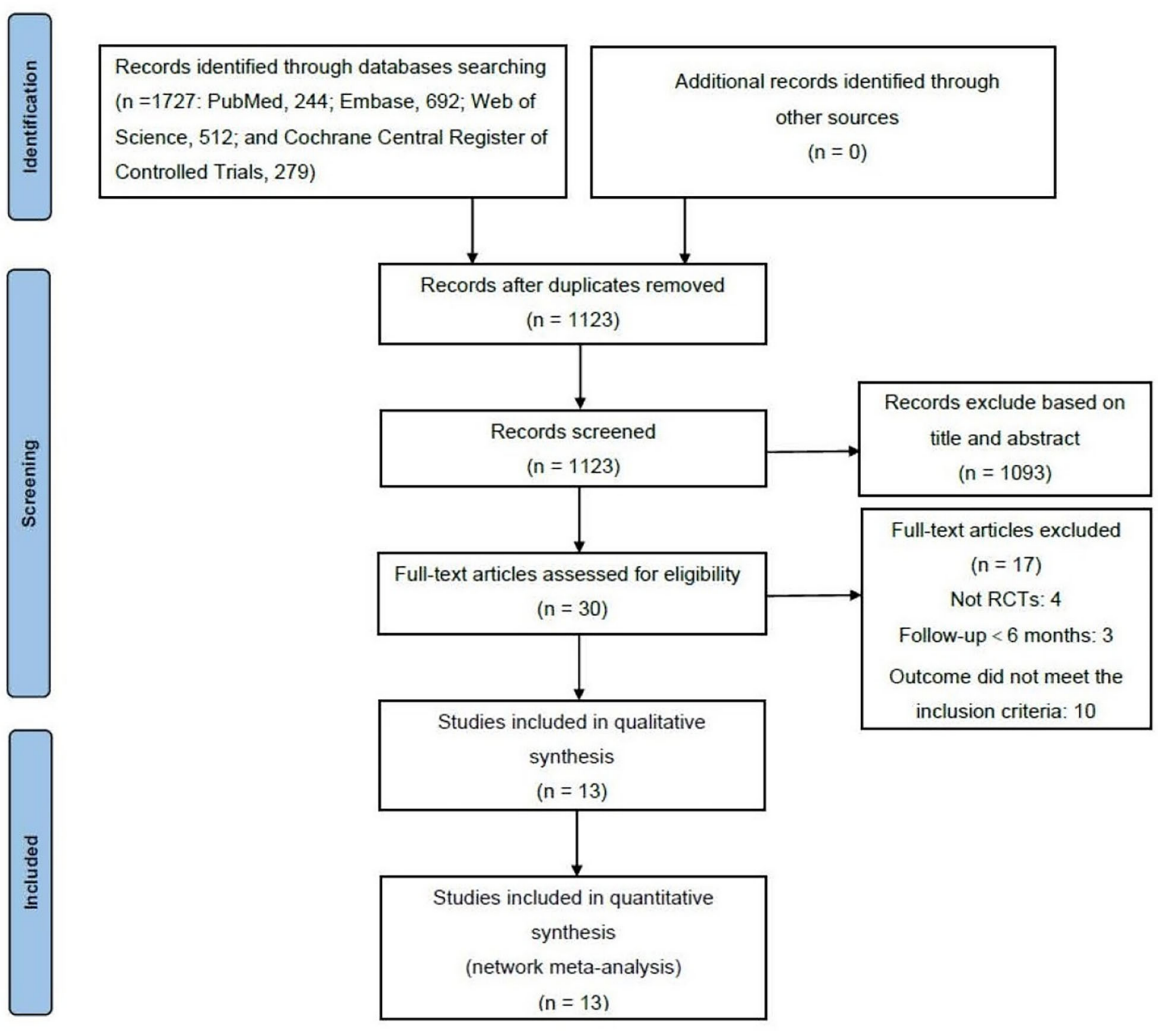
Flow chart depicting the selecting process of included studies.

A total of 1,727 articles (PubMed, 244; Embase, 692; Web of Science, 512; and Cochrane Central Register of Controlled Trials, 279) were retrieved from the electronic databases in the primary search, among which 604 articles were removed for duplicates. After screening the titles and abstracts, 1,093 articles were removed. Thirty full-text articles were reviewed to determine whether they met the inclusion criteria. Eventually, 13 articles were included in the NMA (**Figure 1**) (14, 18, 24–34). Characteristics of all the included RCTs are summarized in **Table 1**. All the RCTs compared two or more interventions and included a total of 2,432 eyes. The mean BCVA and CMT changes from baseline to 6 and 12 months were recorded for the NMA.

**Table 1:** Summary of the characteristics of included studies.

First author	Geographic location	Year	Study design	Intervention	Injection	Follow-up	Total eyes	BCVA-6m	BCVA-12m	CMT-6m	CMT-12m
Tatsumi	Multi-center	2022	RCT	IVA	monthly injection for 3 months, followed by monthly injection based on pro re nata (PRN) regimen	96weeks	25	+4.80 ± 8.45	+7.15 ± 6.9	-83.0 ± 105	-93.0 ± 94.9
				IVA+LASER	ditto		26	+8.65 ± 9.95	+7.55 ± 10.75	-106 ± 158	-115.0 ± 134.7
Li	China	2019	RCT	IVR	3 initial monthly injection, followed by monthly injection based on pro re nata (PRN) regimen until stable vison activity was achieved.	12months	307	+6.7 ± 7.88	+7.8 ± 8.72	-145.1 ± 157.69	-146.5 ± 157.61
				LASER			77	+0.3 ± 11.01	+2.5 ± 8.78	-72.2 ± 153.56	-85.9 ± 166.60
Baker	USA	2019	RCT	IVA	1 injection every 4 weeks	24months	226	NA	+2.1 ± 5.0	NA	-50 ± 55
				LASER			240	NA	+0.1 ± 5.5	NA	-30 ± 69
Lang	Multi-center	2018	RCT	IVR+LASER	4 initial monthly injections followed by pro re nata (PRN) injections	12months	85	+6.4 ± 4.0	+6.5 ± 4.3	NA	-96.7 ± 120.9
				LASER			43	+2.0 ± 3.3	+1.3 ± 3.7	NA	-54.0 ± 89.9
Yang	China	2017	RCT	IVR	1 injection every month. As of month 3, monthly reinjection according to patients’ condition	12months	25	+7.5 ± 6.4	+6.5 ± 6.3	-96 ± 117	-101 ± 112
				IVR+LASER	ditto		28	+7.2 ± 6.1	+7.9 ± 7.1	-108 ± 131	-126 ± 157
Ishibashi	Multi-Center	2015	RCT	IVR	1 injections every month. As of month 3, continue monthly injections if stable vision was not reached.	12months	133	+6.2 ± 7.73	+6.6 ± 7.68	-118.8 ± 161.55	-134.6 ± 131.17
				IVR+LASER	ditto		132	+5.5 ± 8.06	+6.4 ± 10.67	-144.8 ± 166.22	-171.8 ± 160.85
				LASER			131	+0.9 ± 7.81	+1.8 ± 8.27	-35.0 ± 121.98	-57.2 ± 118.60
Berger	Canda	2015	RCT	IVR	3 monthly injections followed by as-needed therapy	12months	75	+7.1 ± 7.83	+8.9 ± 7.83	-129.3 ± 118.69	-143.5 ± 148.25
				IVR+LASER	ditto		73	+5.6 ± 8.58	+8.2 ± 9.44	-114.2 ± 113.29	-152.2 ± 142.47
				LASER			72	+0.9 ± 7.68	+0.3 ± 13.64	-64.4 ± 117.26	-107.1 ± 157.3
Comyn	England	2014	RCT	IVR	3 loading doses of ranibizumab then reinjection every 4 weeks as required	48weeks	22	NA	NA	NA	-131.5 ± 98.0
				LASER			11	NA	NA	NA	-102.9 ± 88.4
Liegl	Germany	2014	RCT	IVR	3 monthly injections and additional injections	12months	32	+7.6 ± 6.7	+6.3 ± 6.5	-88 ± 109	-105 ± 107
				IVR+LASER	ditto		34	+7.2 ± 7.1	+8.4 ± 8.3	-98 ± 197	-129 ± 170
Soheilian	Iran	2012	RCT	IVB	1 injection every 3 months	24months	50	+10.5 ± 10	+10.5 ± 13.5	-36 ± 119	-40 ± 133
				LASER		50	-1 ± 16.5	-1 ± 17	-11 ± 78	+6 ± 86
Mitchell	Multi-Center	2011	RCT	IVR	3 monthly injections at months 0–2, further treatment according to retreatment criteria	12months	116	NA	+6.1 ± 6.43	NA	-118.7 ± 115.07
				LASER			111	NA	+0.8 ± 8.56	NA	-128.3 ± 114.34
				LVR+LASER	ditto		118	NA	+5.9 ± 7.92	NA	-61.3 ± 132.29
Nguyen	USA	2010	RCT	IVR	4 injections at baseline and months 1, 3, and 5	24months	33	+7.24 ± 4.46	+6.61 ± 5.58	NA	NA
				LASER			33	-0.43 ± 4.45	+2.39 ± 4.0	NA	NA
				IVR+LASER	1 injection at month 5		34	+3.8 ± 4.04	+4.81 ± 5.16	NA	NA
Michaelides	England	2010	RCT	IVB	3-9 injections in the first 12 months	12months	42	NA	NA	NA	-130 ± 122
				LASER			38	NA	NA	NA	-68 ± 171

IVA, intravitreal aflibercept; IVB, intravitreal bevacizumab; IVR, intravitreal ranibizumab; LASER, laser, micropulse laser, macular laser, grid laser and focal/grid laser; BCVA: mean change in best corrected visual acuity; CMT: mean change in central macular thickness; NA, Not available.

### Risk of bias assessment

**Figure 2:**
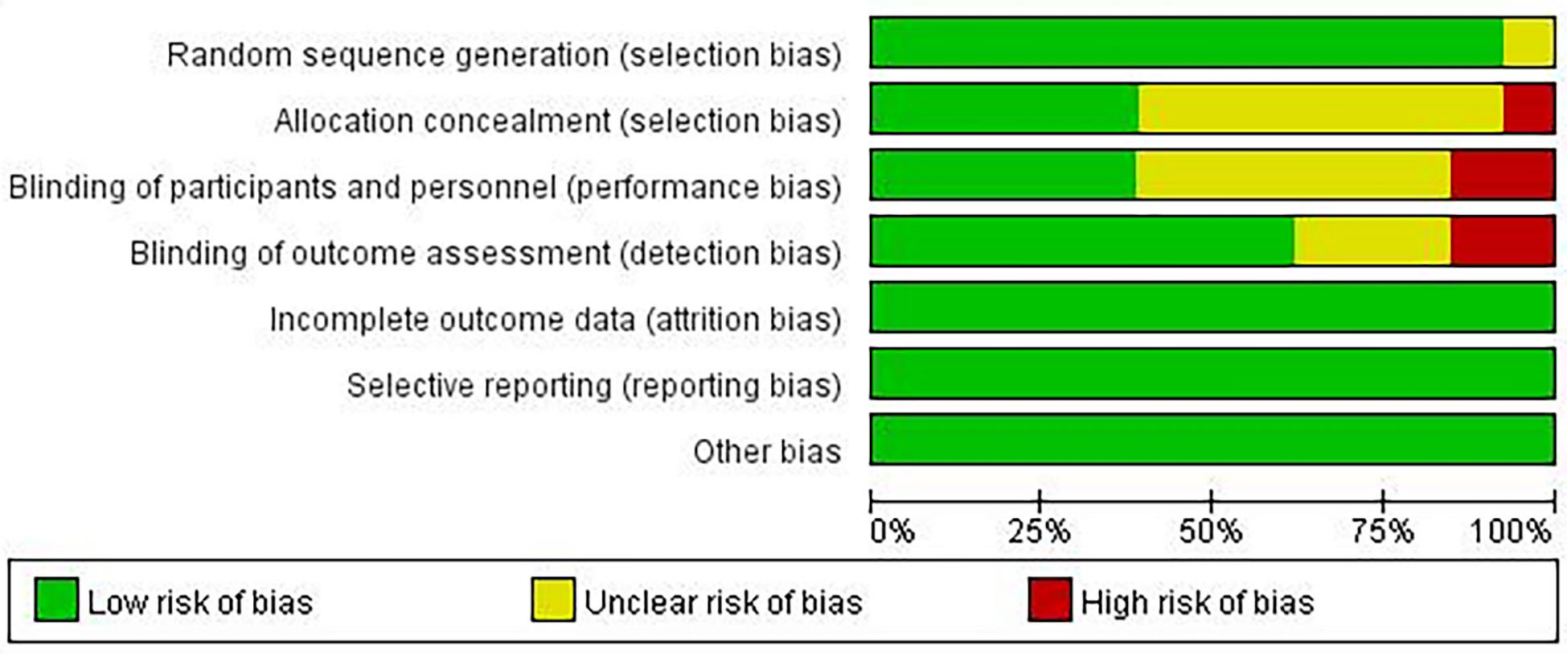
Risk of bias graph.

**Figure 3:**
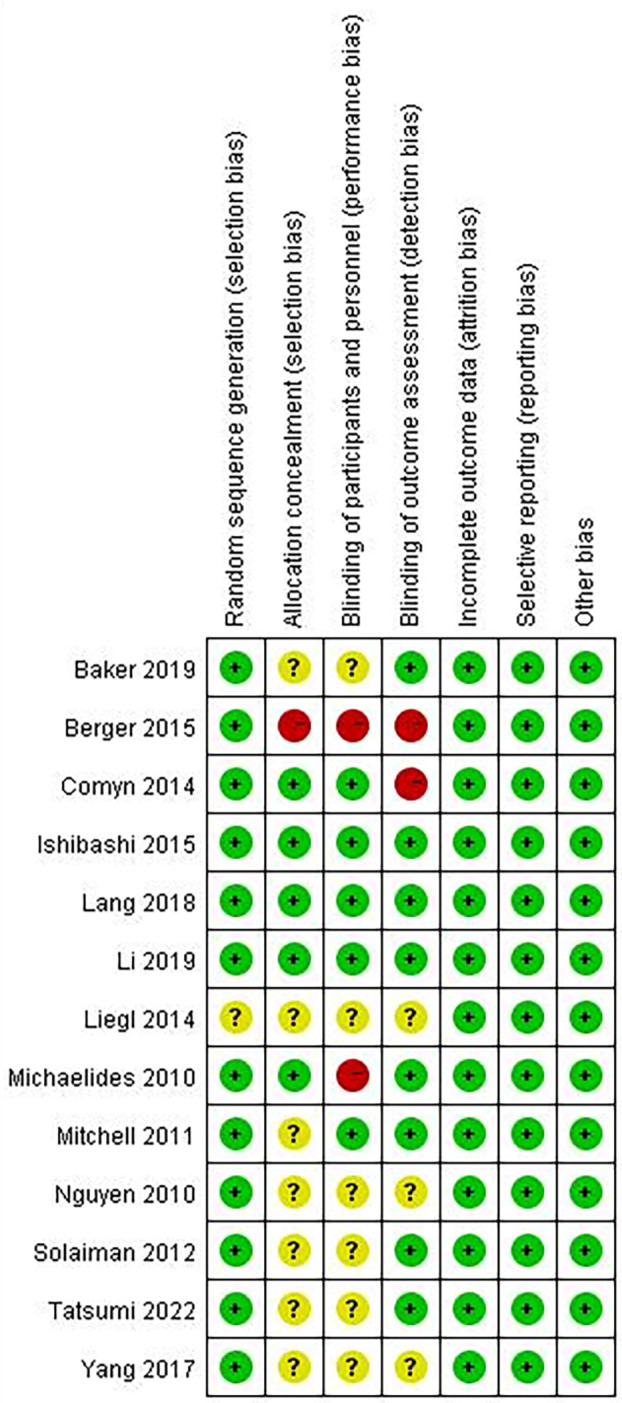
Risk of bias summary.

A summary of the risk of bias assessment for the included studies is shown in **Figures 2**, **3**. One of the studies did not mention the method of generating the random allocation sequence, seven did not mention allocation concealment, six did not mention blinding of participants and personnel, and three did not mention blinding of outcome assessment; therefore, the risk of bias assessment was considered unclear. Additionally, one study had a high risk of bias in the random allocation sequence, one featured a high risk of blinding of participants and personnel, and three featured a high risk of blinding outcome assessment. Overall, quality of the included studies was considered high, although the risk of bias in several studies was high or unclear under some conditions.

### Network meta-analysis

#### Mean BCVA change

**Figure 4:**
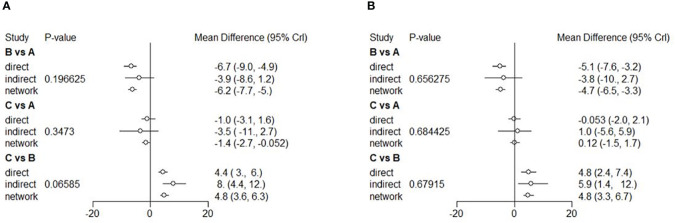
Forest plots of NMA showing mean BCVA change from baseline to 6 **(A)**, and 12 **(B)** months. Different treatments are indicated with capital letters A, B and C in the forest plots. Treatments are indicated as A [anti-VEGF therapy], B [LP therapy], and C [the combined therapy], respectively.

Nine RCTs were included to conduct a NMA for mean BCVA change at six months and 11 RCTs for 12 months. A network of eligible comparisons for the mean BCVA change from baseline to 6 and 12 months is shown in **Figure S1**. Results of the mean BCVA change at six months from baseline suggested that the anti-VEGF group yielded a better vision improvement compared to the combined (MD = 1.5; 95% CI: 0.084, 2.7) and LP (MD = 6.3; 95% CI: 5.1, 7.6) therapies (**Figure 4A**). Likewise, the combined therapy yielded better vision improvement compared to the LP therapy (MD = 4.8; 95% CI: 3.7, 6.3). The results of ranking based on simulations suggested that anti-VEGF therapy (97.715%) was the best, followed by combined (2.285%) and LP (0.000%) therapies (**Figure S7**). However, at 12 months, there was no significant difference between the anti-VEGF and combined therapies (MD = 0.1; 95% CI: -1.7, 1.5). The anti-VEGF (MD = 4.7; 95% CI: 3.3, 6.5) and combined (MD = 4.8; 95% CI: 3.3, 6.7) therapies were significantly superior to the LP therapy (**Figure 4B**). Ranking based on simulations suggested that combined therapy (56.215%) was the best, followed by anti-VEGF (43.785%) and LP (0.000%) therapies (**Figure S8**). All the comparisons showed no significant heterogeneity (p>0.05). However, the I2 statistic showed high heterogeneity when comparing the mean BCVA change at 12 months between the anti-VEGF and LP therapies (**Figures S3**, **4**). Funnel plots on the mean BCVA changes at 6 and 12 months were presented in **Figures S11**, **S12**. Visual inspection showed no significant asymmetry in plots, while Egger’s tests also suggested that no potential threat of publication bias on the mean BCVA changes at 6 months (p=0.935) and 12 months (p=0.532).

#### Mean CMT change

**Figure 5:**
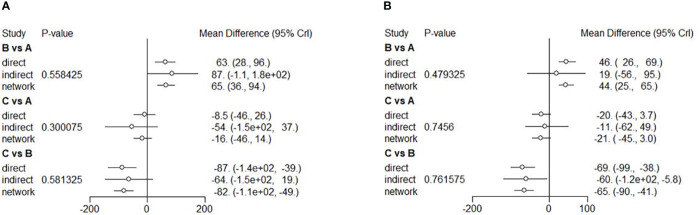
Forest plots of NMA showing mean CMT change from baseline to 6 **(A)**, and 12 **(B)** months. Different treatments are indicated with capital letters A, B and C in the forest plots. Treatments are indicated as A [anti-VEGF therapy], B [LP therapy], and C [the combined therapy], respectively.

Eight RCTs were included to conduct NMA for the mean BCVA change at six months and 12 RCTs at 12 months. A network of eligible comparisons for the mean CMT change from baseline to 6 and 12 months is shown in **Figure S2**. The NMA comparing the combined therapy versus anti-VEGF therapy showed no difference in the mean CMT change between the two therapies (MD = -16; 95% CI: -46, -13). Both anti-VEGF (MD = -65; 95% CI: -93, -37) and combined (MD = -81; 95% CI: -0.011, -50) therapies had a better outcome with a significant change in terms of reduced CMT compared to the LP therapy (**Figure 5A**). Ranking based on simulations suggested that the combined therapy (86.4%) was the best, followed by the anti-VEGF (13.6%) and LP (0.0%) therapies (**Figure S9**). The NMA of mean CMT change at 12 months showed similar results. There was no difference in the mean CMT change between the anti-VEGF and combined therapies (MD = 21; 95% CI: -3.0, -44). Efficacy of the anti-VEGF (MD = -44; 95% CI: -65, -25) and combined (MD = -65; 95% CI: -90, -41) therapies was better than that of the LP therapy (**Figure 5B**). Ranking based on simulations suggested that the combined therapy (95.61%) was the best, followed by the anti-VEGF (4.39%) and LP (0.00%) therapies (**Figure S10**). All the comparisons showed no significant heterogeneity (p>0.05) (**Figures S5**, **6**). Funnel plots on the mean CMT changes at 6 and 12 months were showed in **Figures S13**, **S14**. Visual inspection showed little asymmetry in plots, and Egger’s tests suggested the absence of substantial publication bias on the mean CMT changes at 6 months (p=0.739) and 12 months (p=0.680).

## Discussion

In this NMA, which included 13 studies and a total of 2,422 eyes, we systematically reviewed the published literature and compared the efficacy of three different interventions in patients with DME. It was indicated that compared with the LP therapy, both the anti-VEGF therapy alone and combined anti-VEGF therapy with LP therapy were the most efficacious treatments, with no statistical significance based on the mean CMT change at six and 12 months, as well as the mean BCVA change at 12 months. We found that anti-VEGF therapy alone was better than the combined and LP therapies based on the mean BCVA change at six months. One possible reason is that compared with anti-VEGF therapy alone, the combined therapy may have a stronger anti-angiogenic and anti-inflammatory effect in the early stage after injection, which only affects the decrease in CMT, but has no significant improvement in BCVA (35). Additionally, the adverse effects of LP therapy may provide an explanation for the result that the anti-VEGF therapy alone was better than the combined therapy based on the mean BCVA change at six months. Regarding heterogeneity, we suspect that the high heterogeneity of the mean BCVA change at 12 months was mainly due to the large sample size, but limited therapeutic efficacy of the study by Backer et al. (25).

Although intravitreal injection of anti-VEGF agents has been the standard therapy for DME, LP treatment is still often used (2). LP therapy is associated with severe vision loss (36). With the development of novel LP technologies, these adverse effects have reduced (37). This study involved conventional LP (such as grid LP) and novel LP (such as subthreshold LP) therapies. The NMA was based on the assumption that all LP therapies were same and clinicians should pay attention. However, it is also worth mentioning that conventional LP therapy was reported at least as effective as subthreshold LP therapy in the treatment of DME in the previous meta-analysis (38, 39). Moreover, LP therapy has a significant advantage as a long-lasting treatment compared with anti-VEGF therapy, the latter of which is a short-term treatment (40). Patients need to be followed-up for a long time to monitor therapeutic efficacy, and more long-term outcomes are needed to perform analysis and comparison. Owing to repeated injections, anti-VEGF therapy has complications, including intraocular pressure spikes (41) and endophthalmitis (42), to which attention should be paid during treatment. Therefore, anti-VEGF therapy may not be a good treatment option for all patients. A combination of anti-VEGF and LP can reduce the frequency of injections and thus, may solve this problem.

Previous studies have shown that the combined therapy is more effective (39). However, a recent study indicated that anti-VEGF therapy was the most efficacious based on the mean BCVA and CMT changes at 12 months, while anti-VEGF and combined therapies had no significant difference in the decrease of CMT at six months (43). Further studies are required to provide more evidence. According to the present study, anti-VEGF therapy alone and combined therapy are both worth considering. The choice of treatments should consider the patient’s tolerance, adherence, economic situation, and so on.

These therapies in our network meta-analysis are commonly used for the treatment of patients with DME; therefore, the results of our study will be instructive for clinical treatment. However, this study has several limitations. First, the number of included studies was relatively small, although they were generally high-quality studies. Second, the baseline characteristics of the patients in different studies were not balanced, but they were not included in the NMA models. The intervals between anti-VEGF and LP therapies and the types of LP therapy were also inconsistent. This might have potentially influenced the validity of the results of the mean BCVA and CMT changes. Finally, we did not compare the effects of the different anti-VEGF agents. To evaluate the efficacy of these therapies more accurately, more high-quality RCTs are necessary.

In conclusion, this NMA showed evidence of comparable efficacy in terms of BCVA and CMT between anti-VEGF therapy alone and anti-VEGF combined with LP therapy, with no overall significant difference. Considering the results of the forest plots and ranking based on simulations of treatments and need for long-term treatment, combined therapy is recommended for the treatment of patients with DME.

## Data availability statement

The original contributions presented in the study are included in the article/**Supplementary Material**. Further inquiries can be directed to the corresponding author.

## Author contributions

WQ designed the study and revised the manuscript. JC and HW collected and analyzed the data. JC and HW drafted the manuscript. All authors contributed to the article and approved the submitted version.
