# Supplementary material for: Intravitreal anti-vascular endothelial growth factor, laser photocoagulation, or combined therapy for diabetic macular edema: A systematic review and network meta-analysis
Source: Front Endocrinol (Lausanne). 2023 Feb 2;14:1096105. doi: 10.3389/fendo.2023.1096105 (PMC9933865; doi:10.3389/fendo.2023.1096105)
Supplement: Supplementary file 1 [file DataSheet_1.docx]

Table S1: Search strategy

| **Relevant text of diabetic macular edema** | **Relevant text of laser photocoagulation** |
| --- | --- |
| 1. Diabetic macular edema | 11. Laser |
|  | 12. Photocogulation |
| **Relevant text of anti-VEGFs** | 13. 11 OR 12 |
| 2. Anti |  |
| 3. Vascular endothelial growth factor | **Final strategy** |
| 4. VEGF | 14. 1 AND 10 AND 13 |
| 5. 2 AND 3 OR 2 AND 4 |  |
| 6. Ranibizumab |  |
| 7. Bevacizumab |  |
| 8. Aflibercept |  |
| 9. Pegaptanib |  |
| 10. 5 OR 6 OR 7 OR 8 OR 9 |  |

Pubmed: <https://pubmed.ncbi.nlm.nih.gov/>

Embase: <https://www.embase.com/>

Web of science: <https://www.webofscience.com/>

Cochrane Central Register of Controlled Trials: <https://www.cochranelibrary.com/>


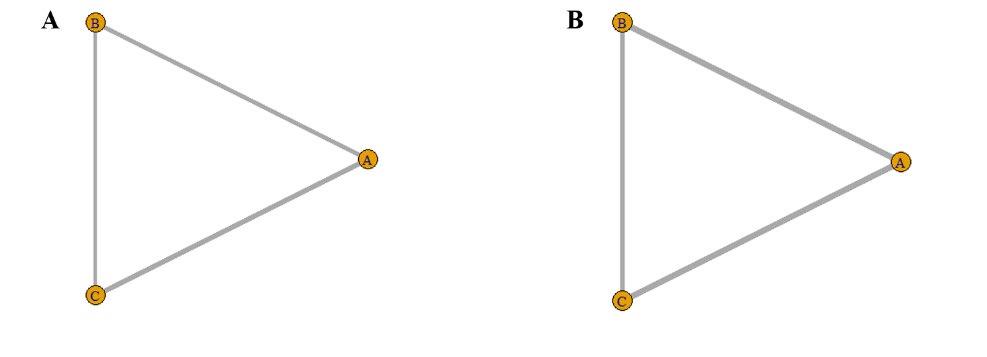


**Figure S1. Network of eligible comparisons for mean BCVA change from baseline to 6 (A), and 12 (B) months.** Treatments are indicated as A [anti-VEGF therapy], B [LP therapy], and C [the combined therapy], respectively.


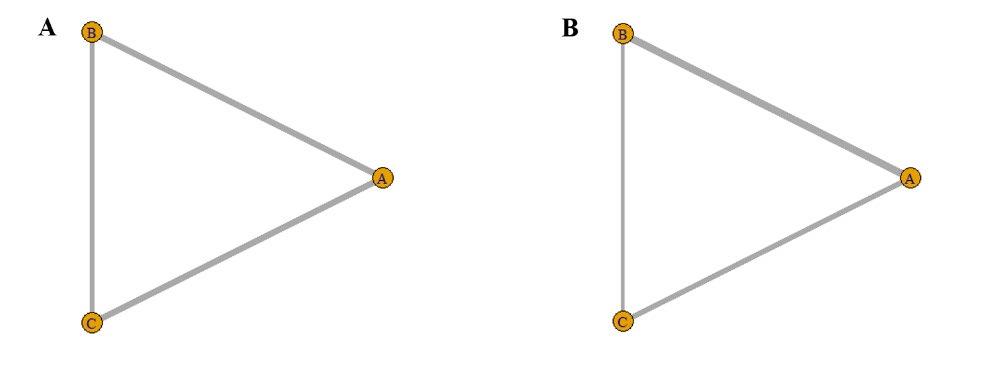


**Figure S2. Network of eligible comparisons for mean CMT change from baseline to 6 (A), and 12 (B) months.** Treatments are indicated as A [anti-VEGF therapy], B [LP therapy], and C [the combined therapy], respectively.


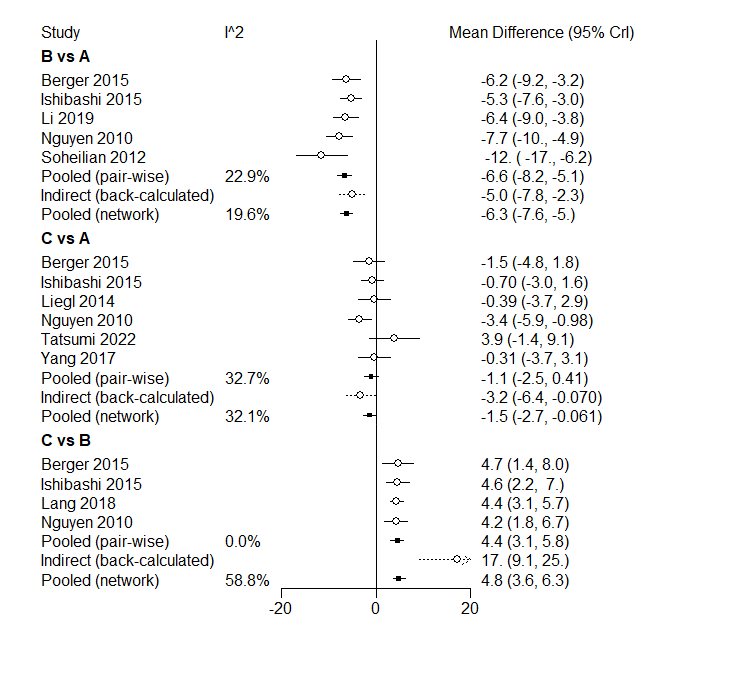


**Figure S3: Forest plots of NMA showing mean BCVA change from baseline to 6 months.** Treatments are indicated as A [anti-VEGF therapy], B [LP therapy], and C [the combined therapy], respectively.


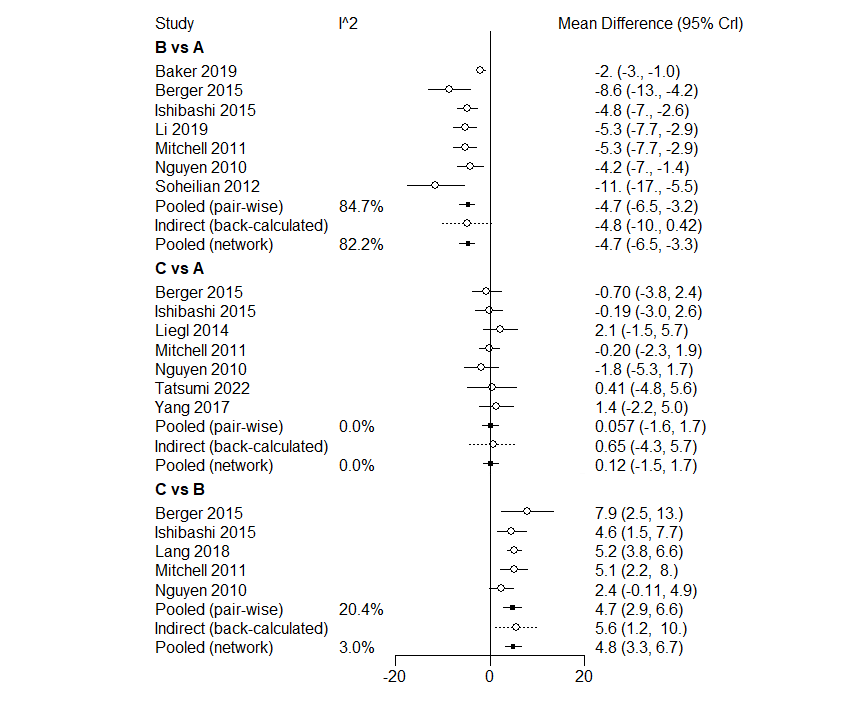


**Figure S4: Forest plots of NMA showing mean BCVA change from baseline to 12 months.** Treatments are indicated as A [anti-VEGF therapy], B [LP therapy], and C [the combined therapy], respectively.


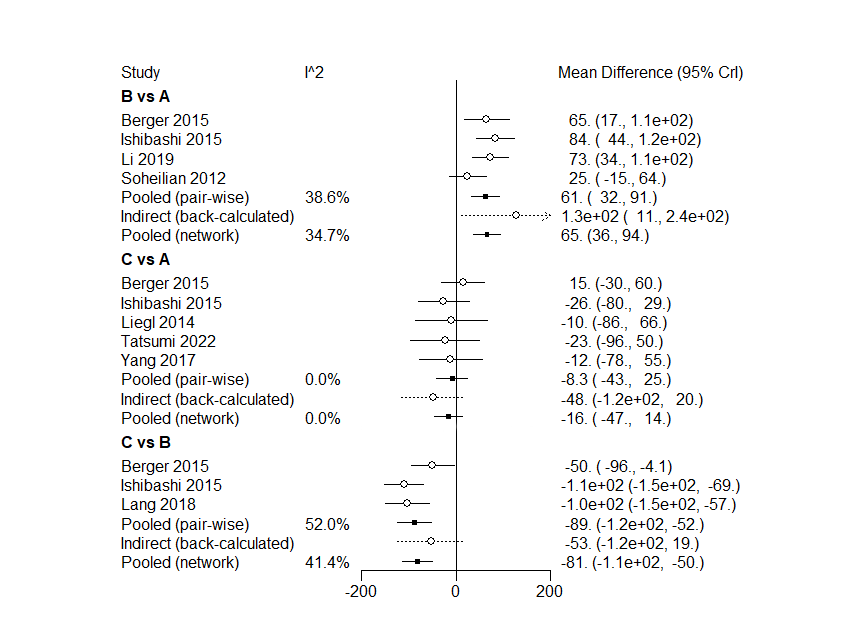


**Figure S5: Forest plots of NMA showing mean CMT change from baseline to 6 months.** Treatments are indicated as A [anti-VEGF therapy], B [LP therapy], and C [the combined therapy], respectively.


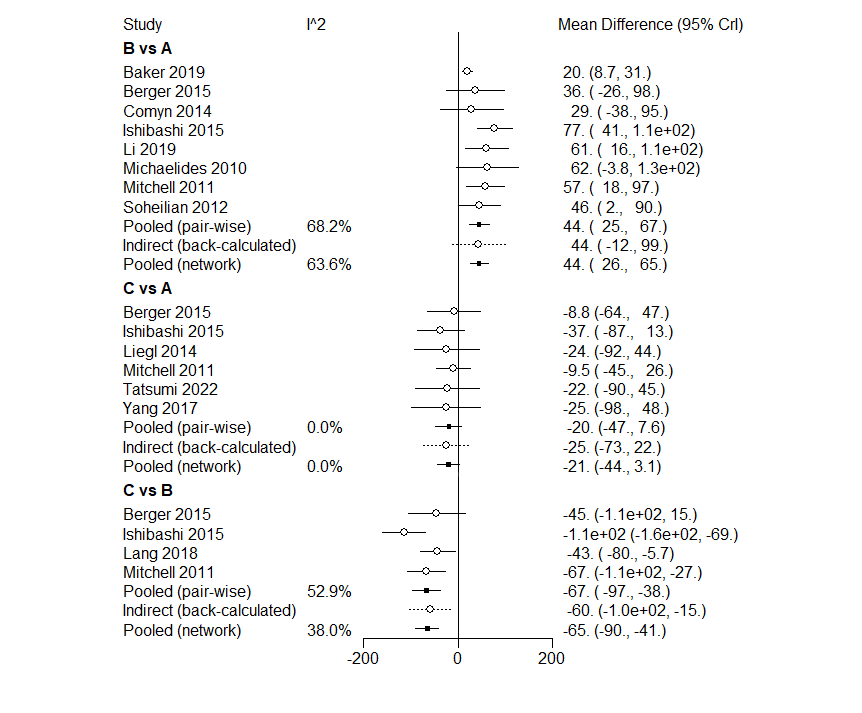


**Figure S6: Forest plots of NMA showing mean CMT change from baseline to 12 months.** Treatments are indicated as A [anti-VEGF therapy], B [LP therapy], and C [the combined therapy], respectively.


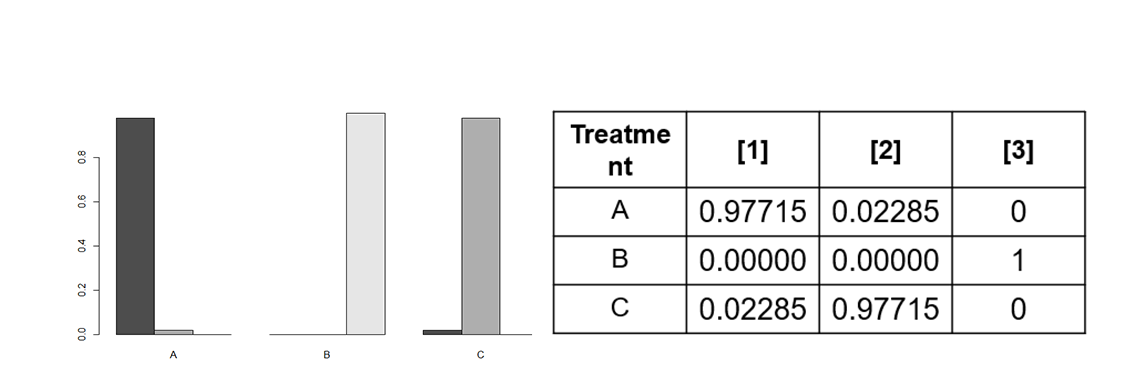


**Figure S7: Ranking based on simulations of treatments for mean BCVA change from baseline to 6 months.** Treatments are indicated as A [anti-VEGF therapy], B [LP therapy], and C [the combined therapy], respectively.


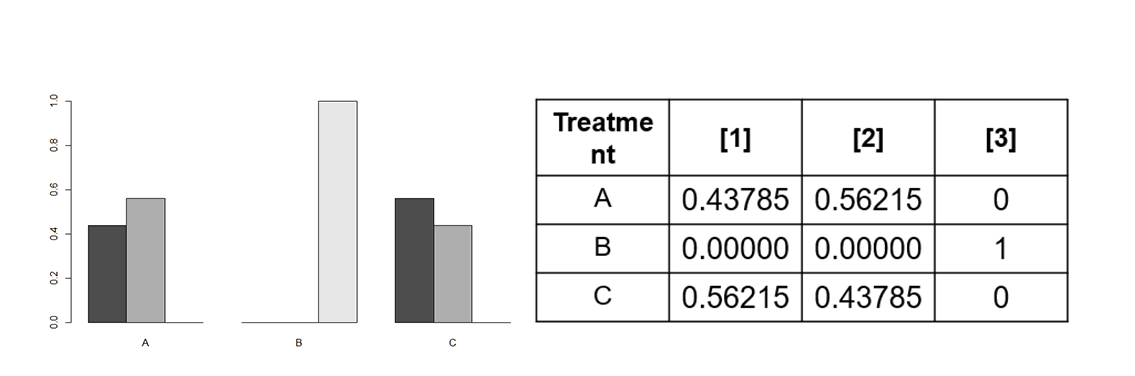


**Figure S8: Ranking based on simulations of treatments for mean BCVA change from baseline to 12 months.** Treatments are indicated as A [anti-VEGF therapy], B [LP therapy], and C [the combined therapy], respectively.


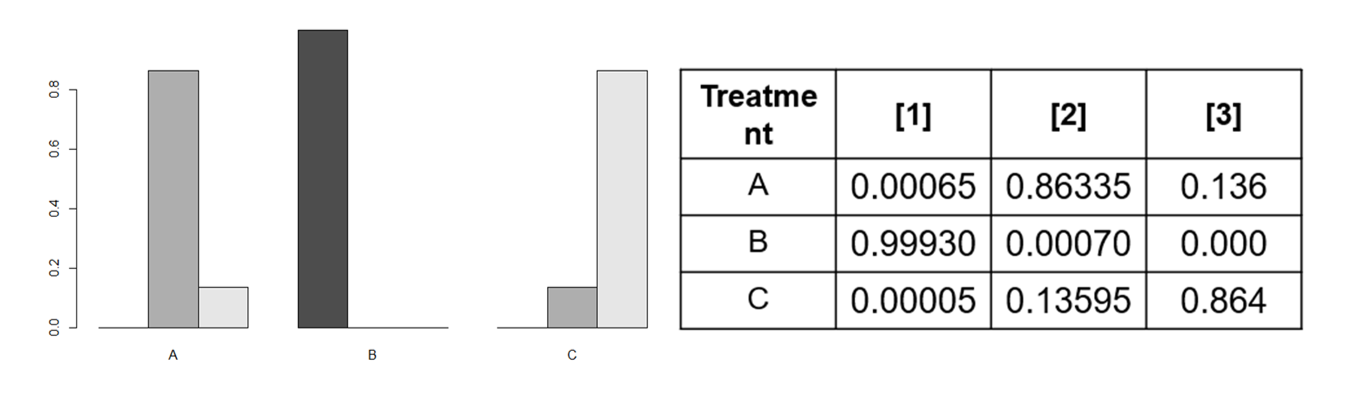


**Figure S9: Ranking based on simulations of treatments for mean CMT change from baseline to 6 months.** Treatments are indicated as A [anti-VEGF therapy], B [LP therapy], and C [the combined therapy], respectively.
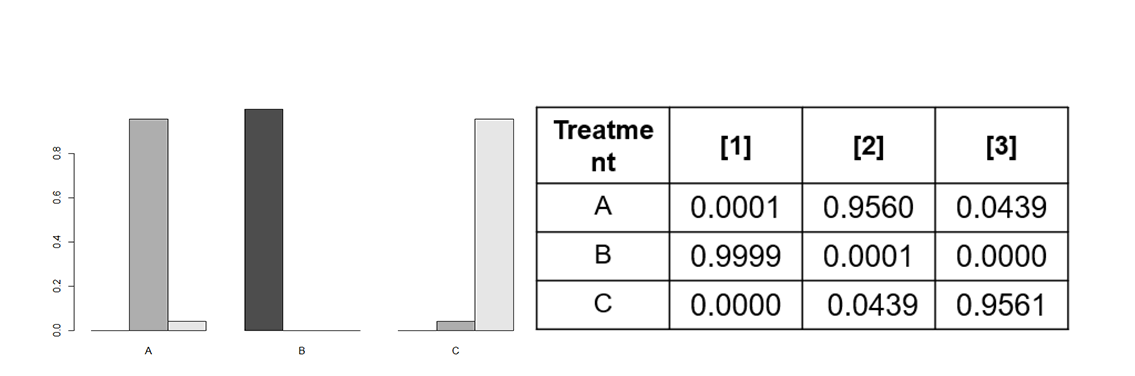


**Figure S10: Ranking** **based on simulations of treatments for mean CMT change from baseline to 12 months.** Treatments are indicated as A [anti-VEGF therapy], B [LP therapy], and C [the combined therapy], respectively.


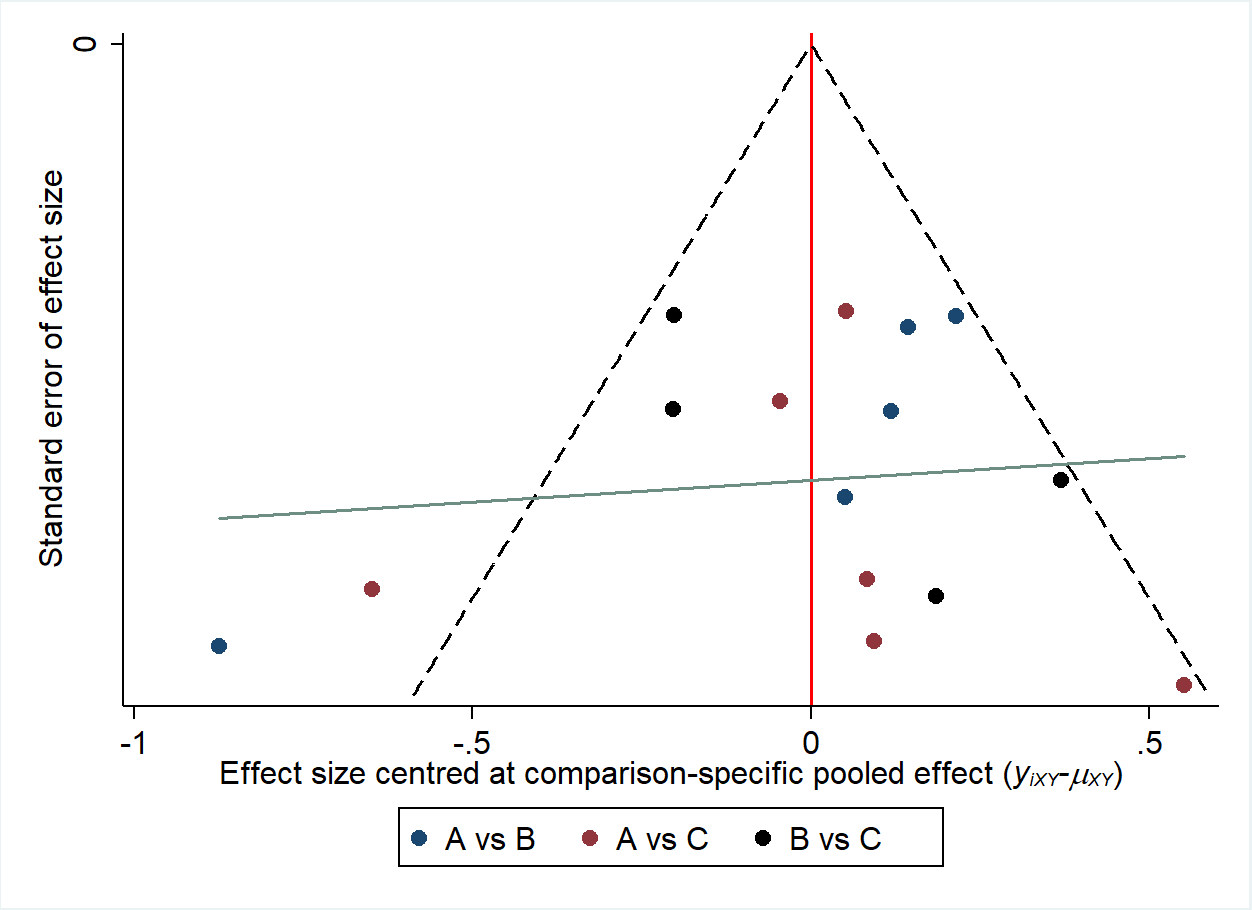


**Figure S11: Funnel plot for the investigating of publication bias on mean BCVA change at 6 months.** Treatments are indicated as A [anti-VEGF therapy], B [LP therapy], and C [the combined therapy], respectively.


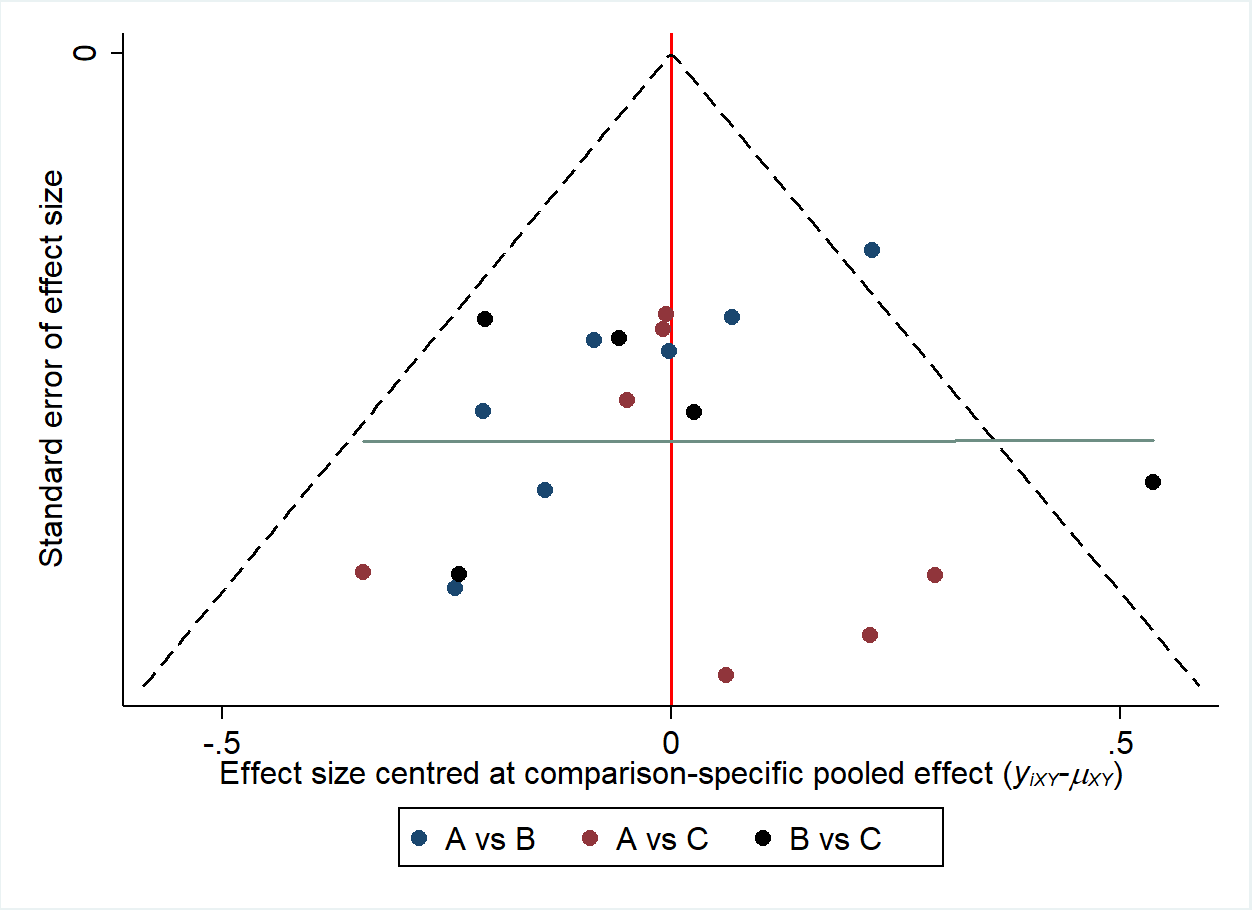


**Figure S12: Funnel plot for the investigating of publication bias on mean BCVA change at 12 months.** Treatments are indicated as A [anti-VEGF therapy], B [LP therapy], and C [the combined therapy], respectively.


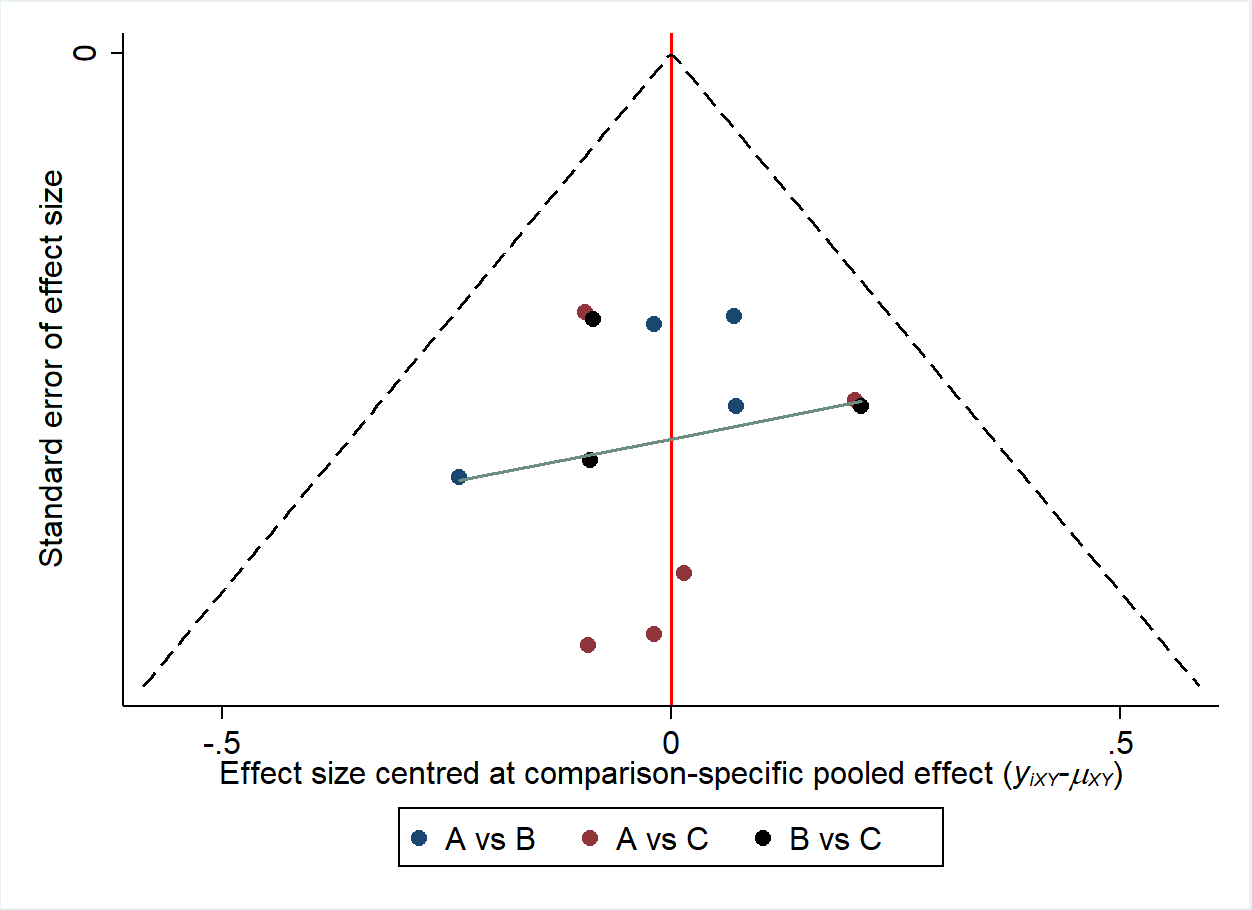


**Figure S13: Funnel plot for the investigating of publication bias on mean CMT change at 6 months.** Treatments are indicated as A [anti-VEGF therapy], B [LP therapy], and C [the combined therapy], respectively.


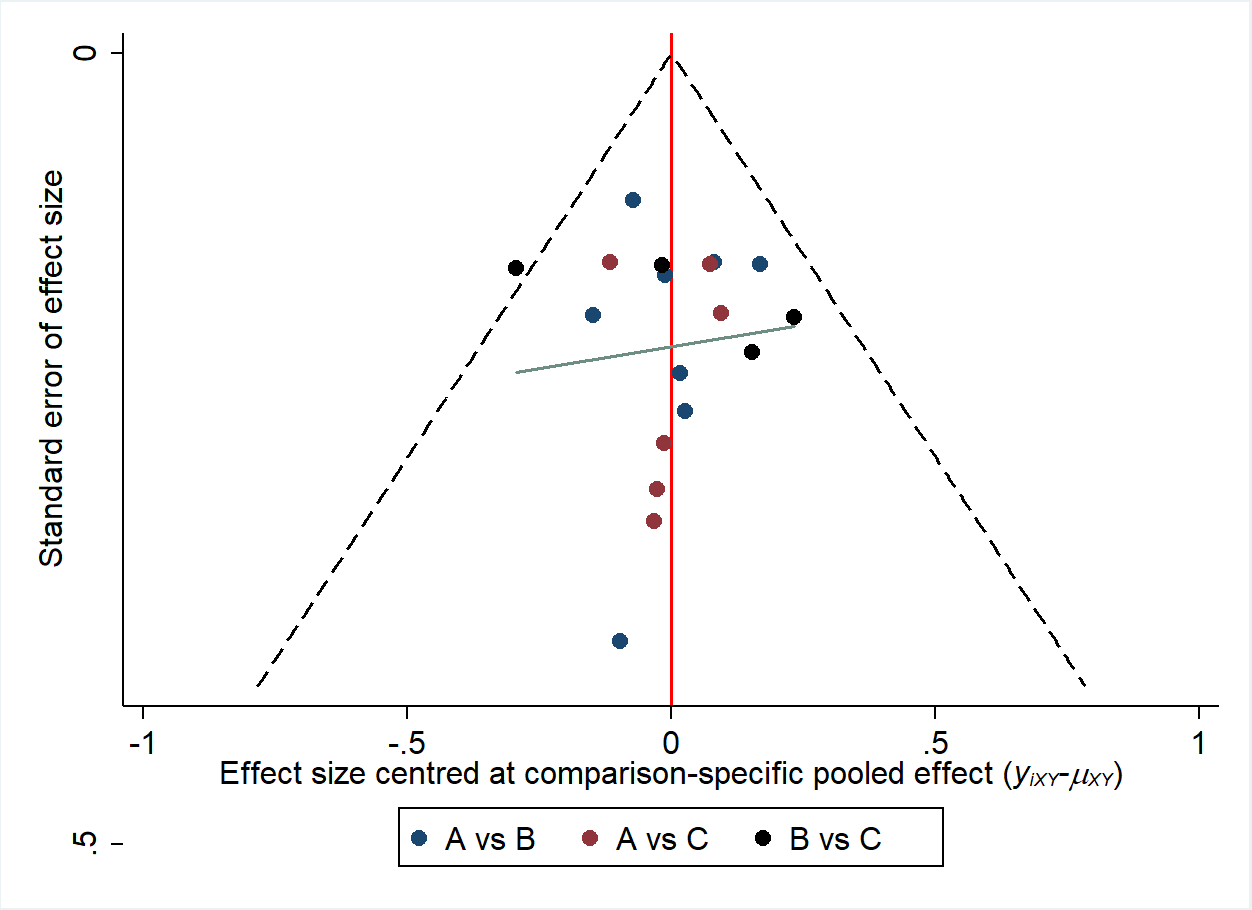


**Figure S14: Funnel plot for the investigating of publication bias on mean CMT change at 12 months.** Treatments are indicated as A [anti-VEGF therapy], B [LP therapy], and C [the combined therapy], respectively.
